# Critical Thoughts About Health Disparities With the NIA Division of Neuroscience

**DOI:** 10.1093/geroni/igab046.1401

**Published:** 2021-12-17

**Authors:** Damali Martin, Cerise Elliott

**Affiliations:** 1 NIA, NIH, Bethesda, Maryland, United States; 2 National Institute on Aging, Bethesda, Maryland, United States

## Abstract

Population-based health disparities studies requires improved research design and appropriate research questions for investigation that will inform evidenced-based interventions and prevention strategies. The NIA Division of Neuroscience is committed to supporting new studies that 1) invests in health priorities as reflected by needs of minoritized populations (e.g. Race/Ethnic minorities; Rural or Sexual Gender Minorities); 2) examines Alzheimer’s Disease and cognitive changes across the individual lifespan; and 3) understands intersectionality of cohorts and minimize potential biases in participant selection. This brief session will outline updated steps to permit critical thought about the use of the NIA’s Health Disparities Framework to examine relevant biological, sociocultural, behavioral and environmental across multiple levels of influence.

